# An Electro–Microbial Process to Uncouple Food Production from Photosynthesis for Application in Space Exploration

**DOI:** 10.3390/life12071002

**Published:** 2022-07-06

**Authors:** Philip J. L. Bell, Ferdinand E. Paras, Sophia Mandarakas, Psyche Arcenal, Sinead Robinson-Cast, Anna S. Grobler, Paul V. Attfield

**Affiliations:** Microbiogen Pty Ltd., Level 4, 78 Waterloo Road, Macquarie Park, NSW 2113, Australia; ferdinand.paras@microbiogen.com (F.E.P.); sophia.mandarakas@microbiogen.com (S.M.); psyche.arcenal@microbiogen.com (P.A.); srobinsoncast@microbiogen.com (S.R.-C.); an.grobler@microbiogen.com (A.S.G.)

**Keywords:** space exploration, life support systems, *Saccharomyces* yeasts, bioregenerative food production

## Abstract

Here we propose the concept of an electro–microbial route to uncouple food production from photosynthesis, thereby enabling production of nutritious food in space without the need to grow plant-based crops. In the proposed process, carbon dioxide is fixed into ethanol using either chemical catalysis or microbial carbon fixation, and the ethanol created is used as a carbon source for yeast to synthesize food for human or animal consumption. The process depends upon technologies that can utilize electrical energy to fix carbon into ethanol and uses an optimized strain of the yeast *Saccharomyces cerevisiae* to produce high-quality, food-grade, single-cell protein using ethanol as the sole carbon source in a minimal medium. Crops performing photosynthesis require months to mature and are challenging to grow under the conditions found in space, whereas the electro–microbial process could generate significant quantities of food on demand with potentially high yields and productivities. In this paper we explore the potential to provide yeast-based protein and other nutrients relevant to human dietary needs using only ethanol, urea, phosphate, and inorganic salts as inputs. It should be noted that as well as having potential to provide nutrition in space, this novel approach to food production has many valuable terrestrial applications too. For example, by enabling food production in climatically challenged environments, the electro–microbial process could potentially turn deserts into food bowls. Similarly, surplus electricity generated from large-scale renewable power sources could be used to supplement the human food chain.

## 1. Introduction

Yeasts have been used in the human food chain since at least the ancient Egyptian and Babylonian times [1,2], and the industrial production of yeast has been established for over 100 years. Due to the widespread applications of *Saccharomyces* spp., they are the largest by volume of all industrially produced microbes on the planet with millions of tonnes of yeast biomass produced per annum for human consumption [2,3]. *Saccharomyces cerevisiae* is traditionally used to produce bread, beer, and wine and has more recently been used as a direct feed supplement in agriculture and aquaculture [4,5,6,7,8]. Due to its long established and widespread use in the human food chain, microbial single-cell protein (SCP) derived from yeast is generally recognized as safe for human consumption.

**Figure 1:**
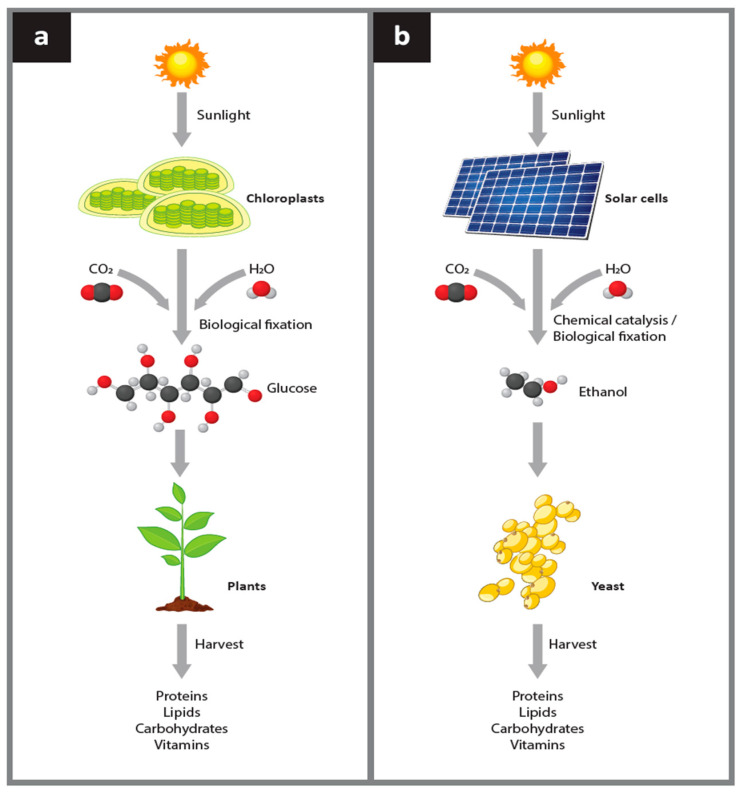
Comparison between plant photosynthesis and the analogous electro–microbial process to produce food. Panel (**a**) Photosynthetic production of food. The initial capture of energy from sunlight by plants uses chlorophyll and other light harvesting pigments located in chloroplasts to absorb photons. The absorbed energy is used in the process of photosynthesis to combine CO_2_ and hydrogen derived from water into sugars. Plant biosynthetic pathways allow the sugar synthesized in photosynthesis to build all amino acids, complex carbohydrates, nucleotides, nucleosides, vitamins, lipids, and other co-factors required to sustain plant growth. The plant biomass is harvested and used by humans or animals as food. Panel (**b**) The electro–microbial production of food. The initial capture of energy from sunlight in the electro–microbial process uses solar panels to absorb photons and generate electrical energy. The electrical energy is used to combine CO_2_ and hydrogen derived from water to form ethanol. Yeast cells then use their biosynthetic pathways to grow on ethanol and synthesize all amino acids, complex carbohydrates, nucleotides, nucleosides, vitamins, lipids, and other co-factors required to sustain yeast growth. The yeast biomass is harvested and used by humans or animals as food.

Currently, all human food is dependent upon plant biomass that is either consumed directly by humans or forms a critical part of the human food chain. Plant growth is based on photosynthesis, which converts carbon dioxide and water into sugar using solar energy (Figure 1a). This process enables plant biomass to be produced under favorable terrestrial climatic conditions. The proposed electro–microbial process to uncouple food production from photosynthesis (Figure 1b) is based on a procedure that is analogous to photosynthesis but it does not attempt to create a semi-artificial or artificial photosynthetic process [9,10]. In this electro–microbial process, energy is captured via solar panels rather than chlorophyll, and this electrical energy is used to synthesize ethanol rather than sugar from carbon dioxide and hydrogen. In either the photosynthetic or electro–microbial processes, the carbon compound ultimately created using the solar energy is used by endogenous plant or microbial biochemical pathways, respectively, to synthesize all the complex organic compounds required for growth of biomass. Both processes can provide human food directly or indirectly via food chains.

Future space exploration such as missions to Mars and the construction of lunar bases will require the development of bioregenerative life support systems (BLSS) [11], which must provide oxygen, water, and food containing all the nutrients required for long-term survival. Lack of any one of these physiological requirements ultimately results in death or sub-optimal health, and so they need to be continuously supplied in any long-term closed ecological system. Nitrogen and phosphates are required for the growth of food sources, and recycled urine could provide these in a closed BLSS system. Adult humans typically excrete about 2 L urine daily, and this contains approximately 10–35 g urea, 0.88 g sodium, 1.68 g chloride, 0.2 g phosphorous, 0.52 g potassium, 0.1 g sulfur, 0.15 g ammonia, plus other ions and molecules in lesser amounts [12,13]. In addition, humans are adapted to live under conditions of relatively low carbon dioxide, and even under mildly elevated levels of carbon dioxide, decision making processes can be adversely affected [14]. For this reason, carbon dioxide must be continuously scrubbed from the air of any BLSS to maintain an atmosphere capable of supporting human life [15].

The use of plants to consume carbon dioxide and produce both oxygen and food for a BLSS has been explored extensively [15,16]. Since 1970, multiple plant growth chamber designs have been used to perform over 50 different plant cultivation experiments with over 40 different species [16]. The mixed results of the studies confirm the environments of the plant growth chambers must be carefully tailored to allow successful plant growth [16]. Challenges include the intrinsic inefficiencies of energy capture in photosynthetic processes [17] and low edible yields of crops leading to optimized crop growth area estimates of greater than 50 square meters per crew member [18,19]. Furthermore, even under ideal conditions, plants typically take weeks to months to produce a crop suitable for human consumption, and even if a wide variety of crops are produced, some essential nutrients, such as vitamin B12, are not synthesized by any plants, only by prokaryotic microbes [20,21].

**Figure 2:**
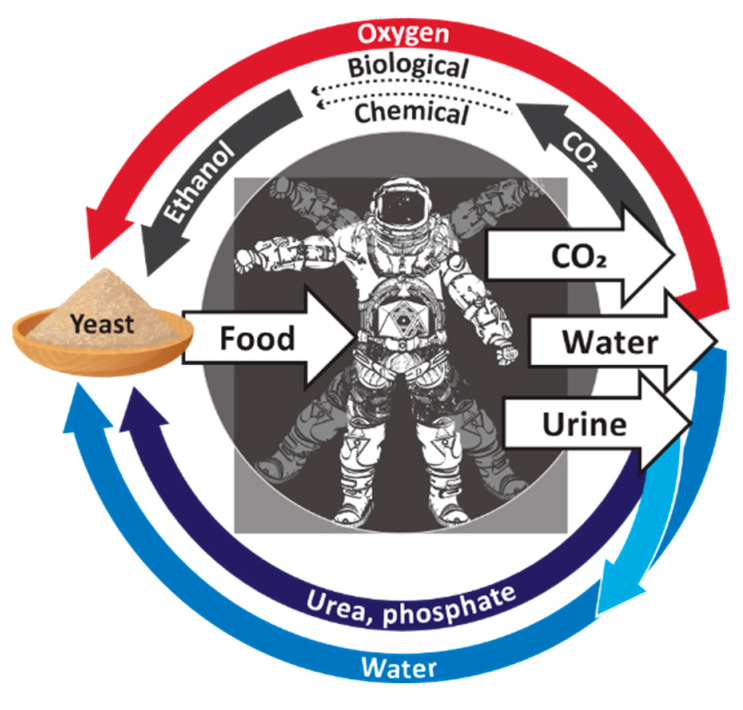
A proposed bioregenerative life support system. Water from a primary source (darker blue arrow) or purified from urine (light blue arrow) is supplied to a fermentor, together with extracted urea and mineral salts (purple arrow). Respired carbon dioxide is converted to ethanol either via chemical synthesis or by using an ethanologenic bacterium (black arrow). Ethanol is fed into the same fermentor as the water, urea, and mineral salts. No other complex organic chemicals are required. Regenerated oxygen (red arrow) is also provided to the fermentor. *Saccharomyces* yeast is inoculated into the fermentor and grows aerobically by utilizing the ethanol, urea, and minerals. The yeast biomass that is produced provides sugars, amino acids, lipids, vitamins, and other nutrient compounds for downstream food production.

Our concept of a microbiologically driven BLSS is shown in Figure 2. A key part of the process is dependent upon technology that uses electricity to produce ethanol from carbon dioxide and water. At least two options are currently available. One option is to use the electricity to hydrolyze water to produce hydrogen, which an ethanologenic bacterium such as *Clostridium ragsdalei* [22] can combine with carbon dioxide to produce ethanol. This technology is relatively mature since the process to convert gas mixtures of CO_2_ and H_2_ into ethanol is already carried out at industrial scale, with companies such as LanzaTech (Skokie, IL, USA) developing the technology to commercially convert waste gas streams into ethanol [23]. The second option is currently at a more developmental stage and uses electricity and chemical catalysts to directly convert carbon dioxide and water into ethanol [24,25]. Once the ethanol has been generated, our proposed process utilizes the biosynthetic capabilities of the yeast *S. cerevisiae* to convert the ethanol into nutritious SCP that could be used for human nutrition.

Although it has long been established that *Saccharomyces* SCP is suitable for both human and animal consumption, a disadvantage of wild-type *Saccharomyces* yeasts for sustainable food production in space is that they require supplementary vitamins and other complex organic compounds to grow efficiently [26,27]. The need for complex media presents difficult economic and technical challenges. To overcome this problem, we used classical selection techniques to generate a strain of *S. cerevisiae* that is optimized to utilize the two-carbon compound ethanol for growth without any added vitamins or other complex organic nutrients. The aim of this paper is to demonstrate the potential for the derived strain of *S. cerevisiae* to grow in aerobic fed-batch culture using ethanol as the sole carbon source, urea for nitrogen, and minimal inorganic salts. We present data showing that yeast biomass grown under these conditions can produce carbohydrates, dietary fiber, lipids, proteins, essential amino acids, and most of the B vitamins required for human nutrition. Using such a yeast, it is possible to conceive of a BLSS module that relies on carbon dioxide scrubbed from the atmosphere and recycled wastes such as urine to provide large quantities of nutritious food on demand, provided a suitable source of electrical energy is available.

## 2. Materials and Methods

### 2.1. Yeast Strain, Fermentation Medium and Bioreactor Culture Conditions

*S. cerevisiae* VITF1 is a non-genetically engineered yeast strain that can grow without addition of exogenous complex organic nutrients such as vitamins. Concentrated medium for fermentation experiments contained (g per L of deionized water), 300 of ethanol, 18.5 of urea, 7.5 KH_2_PO_4_, 3.0 of CaCl_2_.2H_2_O, 15 of MgSO_4_.7H_2_O, 0.297 of Fe_2_(SO_4_)_3_, 0.006 of CuSO_4_, 0.015 of ZnSO_4_, and 0.029 of MnSO_4_. Medium was adjusted to pH 5.0 using KOH. Salts were dissolved and medium sterilized by autoclaving at 121 °C for 15 min. Urea was autoclaved separately and added to the sterile salt solution after cooling. Ethanol was filter-sterilized and added to the cooled medium. Fed-batch fermentations of yeast strain VITF1 were carried out in a continuously stirred, aerated bioreactor controlled at 30 °C, and pH 5.0 ± 0.5 using NaOH and H_2_SO_4_. Concentrated medium containing all the inorganic salts and ethanol was diluted in prewarmed sterile water to give an initial ethanol concentration of 2.5 g per L before yeast cells were inoculated to an equivalent density of 20 g dry cell weight per L of medium. Aeration was maintained by supplying air at flow rate of 2.5 volumes of air per volume of culture per minute, with stirring increasing to a maximum of 900 rpm based on culture demand. An initial batch phase of fermentation was allowed to proceed until ethanol concentration dropped below 0.8 g per L at which point the fed-batch stage was started by pumping fresh medium into the vessel to maintain supply of nutrients. Ethanol concentration in off-gas was monitored by mass spectrometry and the fed-batch phase was controlled using an ethanol feed-back loop maintaining concentration at 0.8 g per L until the propagation was completed. Three fed-batch fermentations were performed. Cells were harvested by centrifugation at 3500× *g* for 20 min and washed three times in deionized water to remove spent medium. Biomass was then dried to <10% moisture using fluidized bed drying [4].

### 2.2. Chemical Analysis of Yeast Biomass

Dried yeast biomass from separate propagations was pooled and assayed for moisture content, dietary fibre, energy, crude protein (amino N × 6.25), amino acids, vitamins, lipids (saturated and unsaturated), ash, and trace elements. Except for trehalose analysis, all analyses were carried out independently by the Australian Government National Measurement Institute, Melbourne, Victoria using official methods of analysis of the Association of Official Analytical Chemists International [28]. Trehalose, a disaccharide of glucose, was extracted by suspending 500 mg yeast cells in 80% volume per volume ethanol and heating in a boiling water bath for 15 min. Sample tubes were then centrifuged at 3000× *g* for 10 min and the supernatant decanted before being evaporated at 60 °C, and the resultant pellet dissolved in deionized water. A 50 µL sample was reacted with trehalase enzyme in 50 mM sodium acetate buffer, pH 4.5, at 50 °C for 30 min. Digested samples were assayed by HPLC against a pure trehalose standard using a Biorad Aminex HPX-87H column equipped with an appropriate guard column. Mobile phase was 4 mM H_2_SO_4_ with a flow rate of 0.6 mL per min, with oven temperature at 35 °C and run time of 25 min.

## 3. Results

### 3.1. Production and Compositional Analyses of Yeast Strain VITF1 Biomass Propagated by Aerobic Growth on Medium Containing Only Ethanol, Urea, and Inorganic Salts without Vitamins

Strain VITF1 grew exponentially on the medium lacking vitamins and other organic micronutrients such as inositol, using ethanol as a sole carbon source and urea as nitrogen source. Yield was 0.45 ± 0.035 g yeast per g of ethanol, productivity was 1.53 ± 0.3 g yeast per L per h, and final cell density was 104 ± 7.5 g per L. Data for dietary fibre, trehalose, energy, protein, ash, and lipid contents of the yeast produced by the fermentation process are shown in Table 1. The dietary fibre component of yeast is composed mainly of beta-1,3- and beta-1,6-linked glucans [29]. The disaccharide trehalose (alpha-D-glucopyranosyl-(1→1)-alpha-D-glucopyranoside) is a major energy storage compound and stress protectant found in yeast [30], and the level accumulated by strain VITF1 is within normal range for *S. cerevisiae* [31]. Total lipids comprised 77% as monounsaturated and 23% as saturated. Monounsaturated lipids were chiefly palmitoleic (C16:1) and oleic (C18:1) acids, and saturated lipids were mostly palmitic (C16:0) and stearic (C18:0) acids. The data shown for lipids are typical of *S. cerevisiae* [32]. The ash component comprised (mg per kg) 25,100 of K, 12,000 of P, 2800 of S, 1970 of Mg, 1500 of Na, 560 of Ca, 440 of Fe, 28 of Zn, 13 of Cu, and 13 of Mn, which agrees reasonably closely with published data [4].

Table 2 gives the amino acid profile of the yeast biomass. The distribution of the various amino acids matches closely to previously published levels for *Saccharomyces* [4]. The vitamin contents of the yeast biomass were (mg per 100 g) 0.27 of thiamine, 2.6 of riboflavin, 23 of niacin, 2.2 of pantothenate, 2.1 of pyridoxine, 0.013 of biotin, and 0.261 of folic acid. These values are in accordance with other published data [33].

### 3.2. Comparison between Nutritional Value of Yeast Strain VITF1 and Recommended Daily Intakes for Active Adult Humans

The observed yield and productivity of strain VITF1 on medium containing only ethanol, urea, and inorganic salts without vitamins indicates that a single, aerated cylindrical fermentor with a working volume of about 3000 L would be capable of converting approximately 240 kg of ethanol into 108 kg of yeast biomass in a 24 h period. Based on chemical analyses of the yeast biomass produced (Table 1), one such fermentor could comfortably produce an estimated 35 kg of protein with all essential amino acids, 13.7 kg of glucose energy in the form of trehalose, 11 kg of lipids, and 41 kg dietary fibre per day. Although dietary requirements of humans vary considerably depending upon age, sex, weight, and energy expenditure, it has been estimated that a typical adult consumes approximately 100 g of protein, 275 g of carbohydrate, 90 g of fat and 25 g of fibre per day [34]. Our results therefore indicate that the 108 kg of strain VITF1 biomass produced could sustainably provide protein for 351 people, glucose-rich carbohydrates for 50 people, lipids for 120 people, and fibre for 1642 people per day. Based on the crude energy content of 1310 kJ per 100 g observed for the yeast (Table 1) and an estimated 13,000 kJ per day requirement for active adult humans [35,36], 108 kg of yeast would provide enough crude energy for approximately 100 people.

Comparison between the recommended daily intakes of essential amino acids and the composition of the yeast (Table 3) indicates that 151 g of yeast per day would be sufficient to exceed the recommended daily intakes for all essential amino acids and that the 108 kg of yeast biomass could provide for 715 people. Thiamine is the limiting vitamin in the yeast biomass (Table 3), but <500 g of yeast per day would provide all the daily requirements for thiamine and more than enough pantothenate, biotin, riboflavin, niacin, pyridoxine, and folate. The 108 kg of yeast produced per day from a 3000 L fermentor would theoretically provide enough vitamins to feed 240 adults.

## 4. Discussion

In this study, we demonstrate that the prototype *S. cerevisiae* strain VITF1 can grow proficiently under aerobic conditions in a medium containing only ethanol, urea, and inorganic salts, with no additions of exogenous vitamins or other complex organic nutrients. When growing on this very minimal medium, VITF1 necessarily synthesizes all its own building blocks and complex components that are required for production of energy, cellular replication, and growth. Since solar energy can be efficiently captured by photovoltaic cells and could be used to fix carbon dioxide into ethanol via either a catalytic route or via a biological route using acetogens, a substantial source of nutrition could be produced without the need to rely solely on growing plant-based foods. Our results indicate that a single 3000 L fermentor could produce >100 kg of yeast biomass per day, which appears sufficient to provide nutrition to support 50 to 100 people per day. This represents a much smaller ‘footprint’ than the hundreds of square meters of area required to provide similar quantities of food using photosynthetic plants [18,19]. Conceptually, large-scale fermentors could also be operated in lunar or planetary life support systems, but smaller bioreactors could be used on orbiting space stations for crews of 10 or fewer people. Other researchers have previously demonstrated that *S. cerevisiae* can grow in microgravity conditions, showing growth rate and viability comparable to yeast grown under normal gravity conditions [41].

Although yeast biomass contains all essential amino acids and a wide range of other nutrients needed for human and animal diets, it seems unlikely that astronauts would thrive on a diet of unprocessed yeast. However, it is noteworthy that *S. cerevisiae-*derived protein is already being processed into commercially available forms that are used as meat and cheese substitutes [42,43]. Furthermore, since yeast biomass has a proven track record as a feed for aquaculture and poultry [6,44,45,46,47], a yeast production facility potentially represents only one module of a functional BLSS, with other BLSS food production modules dedicated to the production of animal proteins using the yeast as a feed.

Analysis of the yeast SCP reveals that yeast biomass produces all essential amino acids and many of the vitamins and other nutrients a human requires in their diets. As shown in Table 3, the yeast SCP is a well-balanced source of essential amino acids for human nutrition with a strong correlation between human recommended daily intakes and the abundance of amino acids in yeast SCP. The vitamins pantothenate, biotin, thiamine, riboflavin, niacin, folate, and pyridoxine were also all synthesized de novo by yeast, and less than 500 g of yeast per day would provide all the daily requirements of these vitamins (Table 3). Whilst yeast do not directly produce vitamin D, it should be noted that they do produce ergosterol, which can be converted to vitamin D using UV irradiation [48]. Though yeast cannot synthesize cyanocobalamin (vitamin B12), if the acetogen route is used to make ethanol, B12 synthesis occurs since it is a cofactor in the Wood–Ljungdahl pathway for fixing carbon dioxide in acetogens [49].

Beyond the nutrients synthesized by yeast, humans also require an exogenous source of vitamin A (beta-carotene), vitamin C (ascorbic acid), vitamin E (alpha-tocopherol), and the essential fatty acids linoleic and alpha-linolenic acid. Since yeast of the genus *Saccharomyces* are a preferred chassis for genetic and metabolic engineering of cell factories [50], it has already been demonstrated that yeast can be individually genetically modified to produce vitamin A [51], vitamin C [52], and vitamin E [53] as well as both linoleic and alpha-linolenic acid [54]. It may therefore be possible in future to engineer a single yeast strain to synthesize all the essential vitamins, amino acids, and fatty acids required for human nutrition from a simple compound such as ethanol. This would be particularly valuable in the context of space exploration since a single organism could synthesize all the nutrients required to sustain human life. However, it is currently unclear whether this desirable goal is possible since the complex pathways required to synthesize these other vitamins could interact with each other and adversely impact growth of a yeast strain such as VITF1 on ethanol as a sole carbon source. Thus, it remains to be determined if it is possible to generate, through synthetic biology techniques, a single yeast strain that can manufacture all types of nutrients including the vitamins and cofactors needed for the human diet under the conditions of growth in this electro–microbial process. An alternative approach would be to grow multiple strains of yeasts (or other microbes) specializing in different nutrient features that can be blended to meet all human dietary needs.

The growth of yeast in space or in BLSS will obviously present challenges. Whilst yeast cells can grow efficiently in microgravity conditions [41], it does appear that their lifespan is shortened [55]. However, targeted deletion of certain genes can restore lifespan. There are also morphological changes and altered gene expression patterns in yeast undergoing growth in microgravity conditions [56]. Nevertheless, *Saccharomyces cerevisae* is a very well understood microbe at the levels of molecular genetics, cell biology, and metabolism and can be optimized by classical genetics, molecular genetic modifications, and adaptive evolution approaches. It is remarkably resistant to physicochemical stresses, including temperature (heat and freezing), desiccation, hyper- and hypo-osmotic pressures, ionic stresses, hydrostatic pressure, nutrient starvation, and acidification, and it has a heightened antioxidant defense system [57,58]. Indeed, yeast biomass necessarily withstands various challenges in concert during its production and use in food and fermentation applications on Earth [58,59]. For example, dried yeast, which is widely produced for the baking, brewing, winemaking, and distilling industries, is produced under conditions that are deliberately stressful (such as high temperatures and high salt concentrations) to induce high levels of trehalose (>20%) that allow the yeast to be desiccated and packed under vacuum and retain viability for many years [4,26,58]. These features would be important for yeast under space or BLSS conditions. Furthermore, it is quite feasible that adaptive evolution can be employed to derive strains that grow efficiently under conditions prevailing in a BLSS, or on a space station. In addition to its potential application in space missions, the ability to decouple human food production from photosynthesis may also provide the opportunity to increase the resilience of the human food chain on Earth. Yeast SCP is employed in the human food chain, where it is currently used in bread and beer and to supplement vegan diets, and it is also converted into extracts or fractionated to isolate and concentrate components such as proteins, carbohydrates, and vitamins for use in a wide range of human food. *Saccharomyces* yeast has also been used in animal feed applications for over 100 years, where it improves growth performance of a wide range of animals [4,5,6,7,8,44,45,46,47,60]. The proven track record of whole-cell yeast biomass as a food source for aquaculture and poultry shows that SCP produced by strains such as VITF1 could be widely employed as a supplement in the human agricultural food chain. Since the yeast described in this study has been optimized using non-GM methods and thus retains its “generally recognized as safe” status, biomass derived from it could be immediately used in animal feed applications without major sociopolitical regulatory issues. Furthermore, the proposed electro–microbial route to produce food is, in theory, resistant to increasing climate change impacts such as droughts, fires, floods, hurricanes, etc. For example, it should be possible to build solar farms in desert regions as part of a solar/hydrogen economy and use the proposed electro–microbial process to provide a reliable, high-quality food source that is produced indoors in fermentors. Since deserts have high rates of insolation and the proposed process does not require a climate suitable to grow crops, it may be particularly advantageous to apply this technology to arid desert regions and, in so doing, convert deserts into food bowls.

## Figures and Tables

**Table 1:** Compositional data for *S. cerevisiae* strain VITF1 grown on ethanol, urea, and inorganic salts without vitamin additions.

Component	Amount (g per 100 g of Dry Yeast)
Dietary fibre	38
Trehalose	12.7
Energy *	1310
Protein (amino N × 6.25)	32.5
Ash	7.0
Total lipids	10

* kJ per 100 g dry yeast.

**Table 2:** Amino acid profile of *S. cerevisiae* strain VITF1 grown on ethanol, urea, and inorganic salts without vitamin additions.

Amino Acid	Amount (mg per kg Dry Yeast)
Aspartic acid	35,000
Serine	18,000
Glutamic acid	56,000
Glycine	14,000
Histidine	7400
Arginine	15,000
Threonine	18,000
Alanine	19,000
Proline	14,000
Tyrosine	10,000
Valine	15,000
Lysine	26,000
Isoleucine	13,000
Leucine	23,000
Phenylalanine	13,000
Methionine	4700
Hydroxyproline	93
Taurine	<50
Cysteine	5800
Tryptophan	3400

**Table 3:** Comparison between the essential amino acids and vitamins profiles of *S. cerevisiae* strain VITF1 and the recommended daily intakes for adult humans.

Nutrient	Nutrient (mg per 100 g Dry Yeast)	RDI (mg) *	Yeast (g per Day to Meet RDI)	Number of People 108 kg Yeast Could Support
**Amino acids**				
Lysine	2600	3040	117	923
Histidine	740	1120	151	715
Threonine	1800	1600	89	1213
Cysteine + Methionine	1050	1520	145	745
Valine	1500	1920	128	844
Isoleucine	1300	1520	117	923
Leucine	2300	3360	146	740
Phenylalanine + Tyrosine	2300	2640	115	939
Tryptophan	340	400	118	915
**Vitamins**				
Pantothenate	2.2	5	227	475
Biotin	0.013	0.030	231	468
Thiamine	0.27	1.2	444	243
Riboflavin	2.6	1.3	50	2160
Niacin	23	16	70	1553
Pyridoxine	2.1	1.3	62	1742
Folate	0.26	0.4	154	702

* RDI refers to recommended daily intake of amino acids [37,38], and vitamins [39,40] A body weight of 80 kg and moderate activity was assumed in calculating amino acid requirements.

## Data Availability

All relevant data are contained within this publication.
